# Machine Learning Models of Polygenic Risk for Enhanced Prediction of Alzheimer Disease Endophenotypes

**DOI:** 10.1212/NXG.0000000000200120

**Published:** 2024-01-10

**Authors:** Nathaniel B. Gunter, Robel K. Gebre, Jonathan Graff-Radford, Michael G. Heckman, Clifford R. Jack, Val J. Lowe, David S. Knopman, Ronald C. Petersen, Owen A. Ross, Prashanthi Vemuri, Vijay K. Ramanan, for the Alzheimer's Disease Neuroimaging Initiative

**Affiliations:** From the Departments of Radiology (N.B.G., R.K.G., C.R.J., V.J.L., P.V.), Neurology (J.G.-R., D.S.K., R.C.P., V.K.R.), and Quantitative Health Sciences (R.C.P.), Mayo Clinic Rochester, MN; and Departments of Quantitative Health Sciences (M.G.H.), Neuroscience (O.A.R.), and Clinical Genomics (O.A.R.), Mayo Clinic Florida, Jacksonville.

## Abstract

**Background and Objectives:**

Alzheimer disease (AD) has a polygenic architecture, for which genome-wide association studies (GWAS) have helped elucidate sequence variants (SVs) influencing susceptibility. Polygenic risk score (PRS) approaches show promise for generating summary measures of inherited risk for clinical AD based on the effects of *APOE* and other GWAS hits. However, existing PRS approaches, based on traditional regression models, explain only modest variation in AD dementia risk and AD-related endophenotypes. We hypothesized that machine learning (ML) models of polygenic risk (ML-PRS) could outperform standard regression-based PRS methods and therefore have the potential for greater clinical utility.

**Methods:**

We analyzed combined data from the Mayo Clinic Study of Aging (n = 1,791) and the Alzheimer's Disease Neuroimaging Initiative (n = 864). An AD PRS was computed for each participant using the top common SVs obtained from a large AD dementia GWAS. In parallel, ML models were trained using those SV genotypes, with amyloid PET burden as the primary outcome. Secondary outcomes included amyloid PET positivity and clinical diagnosis (cognitively unimpaired vs impaired). We compared performance between ML-PRS and standard PRS across 100 training sessions with different data splits. In each session, data were split into 80% training and 20% testing, and then five-fold cross-validation was used within the training set to ensure the best model was produced for testing. We also applied permutation importance techniques to assess which genetic factors contributed most to outcome prediction.

**Results:**

ML-PRS models outperformed the AD PRS (*r*^2^ = 0.28 vs *r*^2^ = 0.24 in test set) in explaining variation in amyloid PET burden. Among ML approaches, methods accounting for nonlinear genetic influences were superior to linear methods. ML-PRS models were also more accurate when predicting amyloid PET positivity (area under the curve [AUC] = 0.80 vs AUC = 0.63) and the presence of cognitive impairment (AUC = 0.75 vs AUC = 0.54) compared with the standard PRS.

**Discussion:**

We found that ML-PRS approaches improved upon standard PRS for prediction of AD endophenotypes, partly related to improved accounting for nonlinear effects of genetic susceptibility alleles. Further adaptations of the ML-PRS framework could help to close the gap of remaining unexplained heritability for AD and therefore facilitate more accurate presymptomatic and early-stage risk stratification for clinical decision-making.

## Introduction

Genetic factors are estimated to explain approximately 71 percent of the risk of Alzheimer's disease (AD).^1,2^ The genetic influences of AD are widely presumed to be polygenic in nature, with numerous genetic factors acting collectively to influence susceptibility.^3^ The apolipoprotein E ε4 allele (*APOEe4*) is the strongest known genetic risk factor of sporadic AD and is associated with increased risk by a factor of 2–3 with 1 allele copy and by a factor of 8–12 with 2 copies.^4^ Genome-wide association studies (GWAS) of clinically diagnosed AD dementia have identified numerous other susceptibility variants, though most of the sequence variants (SVs) implicated have displayed modest individual effect sizes.^5-7^ For a highly heritable disease, there remains great interest in developing tools to characterize the polygenic risk architecture underlying AD more fully to guide clinical risk stratification.

Polygenic risk score (PRS) methods have shown promise for characterizing this complex genomic background.^8,9^ These methods generate a summary measure of risk based on an individual's relevant genetic profile (i.e., their genotypes at known susceptibility variants across the genome). Many methods for PRS calculation exist.^8^ Most standard PRS approaches calculate the combined sum of effect sizes for risk variants taken from GWAS data sets applied toward the dosages of those variants in an individual. In theory, this approach facilitates aggregating the effects of *APOEe4* and other AD susceptibility alleles into a quantitative measure, which can be related to outcomes of interest. For AD, PRS approaches have shown strong associations of risk with clinically diagnosed AD dementia,^9-11^ cognitive decline,^12,13^ and important disease endophenotypes such as amyloid and tau burden.^14,15^

A limitation of standard PRS methods is that they are fundamentally linear. The weighted sum of risk alleles and their effects does not include nonlinear genetic effects such as epistatic (gene × gene interaction) influences.^16^ For example, we cannot expect that the combined effect of SV A and SV B is equal to the sum of the effects of SV A and SV B when considering the activation of biological pathways. Moreover, weights for risk variants drawn from GWAS^5-7^ reflect effects across the population and therefore may underestimate clinical subpopulations where specific combinations of gene variants may be driving susceptibility. Potential gene-gene interaction terms prove difficult to compute because generating weights for them presupposes the relationship between the constituent SVs. Therefore, improvements that address these limitations of existing PRS approaches may better explain the heterogeneity of AD and thus have greater clinical utility.^17^

Previously, machine learning (ML) methods have been used to assign functional annotations to genes, as well as to understand mechanisms of gene expression by modeling interactions within a network framework.^18^ In addition, ML models have shown usefulness in drawing novel insights from multiomics data, including for cancer^19^ and other non-AD traits.^20^ We hypothesized that ML models (especially those accounting for nonlinear interactions) would outperform standard PRS for prediction of important AD endophenotypes. Furthermore, we hypothesized that established approaches for understanding ML models would illuminate the relative importance of individual SVs and SV × SV interactions toward model prediction to provide insights on underlying disease pathways and mechanisms.

## Methods

### Participant Selection and Description

The Mayo Clinic Study of Aging (MCSA) is a population-based prospective study of older adults residing in Olmsted County, Minnesota.^21^ Individuals were identified for recruitment using the Rochester Epidemiology Project medical records linkage system.^22^

The Alzheimer's Disease Neuroimaging Initiative (ADNI) is a longitudinal multicenter study to facilitate development of clinical, imaging, genetic, and biochemical biomarkers for the early detection and tracking of AD.^23,24^ Individuals were recruited from more than 50 sites across the United States and Canada. Further information about the ADNI can be found at adni.loni.ucs.edu/.

Data from the MCSA and ADNI were harmonized where applicable and combined for analyses to maximize statistical power and provide the data set with most dynamic range. The MCSA alone, due to its nature as an epidemiologic study, does not have a large proportion of cases with cognitive impairment. This flaw is rectified by combination with the ADNI data set, which is recruited as a more traditional prospective study with a structure similar to clinical trials. Primary inclusion criteria included the presence of genome-wide SV genotype data and cross-sectional amyloid PET data. In addition, participants of the MCSA study were assigned a clinical diagnosis by consensus approach, which was applied to differentiate cognitively unimpaired vs cognitively impaired (i.e., mild cognitive impairment or dementia) individuals for use as a complimentary outcome of interest.

#### MCSA Genomic Data

Genomic DNA were extracted from stored peripheral blood samples for MCSA participants. A “GWAS backbone” data set was generated through a custom automated workflow for genotyping-by-sequencing (GxS) developed at the Regeneron Corporation [Tarrytown, NY]. The GxS workflow generated a forced call set for specific 1000 Genomes project 30x biallelic variants in regions intended to capture common tagging variation. Variant sites with zero depth were removed. Postsequencing quality control included checks for gender discordance, duplicate samples, and contamination >5% estimated using VerifyBamID.

Genetic ancestry analyses used a subset of 4,874 high-quality SVs that overlapped with HapMap3 SVs. A kernel density estimator approach was applied to principal component analysis (PCA) trained on individual HapMap3 populations to generate ancestry assignments. To limit confounding, analyses were restricted to individuals with European ancestry, and PCA eigenvectors of ancestry were generated for use as covariates in regression and ML modeling.

Assessments for cryptic relatedness were performed using identity-by-descent analysis in PLINK, version 1.9.^25^ Relatedness analyses were performed subsetting to high-quality common SVs, with minor allele frequency (MAF) > 10% and genotype call rate >95%. Overly related samples were removed based on a conservative threshold of PI HAT ≥0.1875.

The processed GxS data were then subjected to additional quality control, including exclusion for SV genotyping rate <95%, sample genotyping rate <98%, monomorphic SVs, or Hardy-Weinberg equilibrium p < 1 × 10^−6^. This resulted in 1,352,354 SVs available for 5,092 MCSA participants with European ancestry. Imputation was performed on this set using the TOPMed Imputation Server^26,27^ and TOPMed GRCh38/hg38 build reference panel. Monomorphic variants and SVs with low imputation quality (*r*^2^ < 0.8) were removed. This resulted in a total of 31,371,642 SVs, of which 8,094,342 had MAF >1%.

#### ADNI Genomic Data

For ADNI participants, GWAS array data were acquired and filtered for standard quality control metrics, as previously described.^28,29^ Processed genotype files for participants from the various ADNI study phases were downloaded from the LONI web data sharing platform. Overly related samples were removed as previously described.^15^ Genome-wide imputation using the TOPMed Imputation Server (with identical protocols to the MCSA set) was performed separately within each batch (by GWAS array) and then merged. This resulted in 16,502,548 variants (8,054,769 with MAF ≥1%) for 1,661 individuals within the ADNI data set.

#### Neuroimaging Data

In the MCSA, amyloid PET scans were created using an in-house fully automated image-processing pipeline, described here.^30^ Imaging was performed with Pittsburgh compound B (PiB).^31^ For each scan, a standardized uptake value ratio (SUVR) measure of global cortical amyloid burden was generated by normalizing median tracer uptake across a collection of defined regions of interest (prefrontal, orbitofrontal, parietal, temporal, anterior cingulate, and posterior cingulate/precuneus) to the cerebellar crus gray matter.

In the ADNI, amyloid PET was performed with ^18^F-florbetapir (AV-45) using acquisition and processing protocols as described at adni-info.org and with summary measures of global cortical amyloid load downloaded from the ADNI database.^32^

We used the Centiloid (CL) scale to harmonize measurements of amyloid PET burden across the MCSA and ADNI data sets. Conversion from SUVR units to the CL scale was performed as recommended^33^ and as used in previous publications.^30^ In brief, the CL scale ranges from 0 to 100, anchored by amyloid-negative controls younger than 45 years at the low end (average CL value of zero) and patients with typical Alzheimer dementia at the high end (average CL value of 100). The level-1 stepwise approach defined in a previous study^33^ allows for conversion of PiB-PET SUVR measures to CL values, while level-2 approaches facilitate conversion of other amyloid PET tracer SUVR measures to CL values. The primary outcome for our analyses was amyloid PET CL as a continuous measure. As a secondary outcome, amyloid PET positivity was defined by CL ≥ 17.^34^

### Statistical Methods

#### Data Set Creation

**Figure 1:**
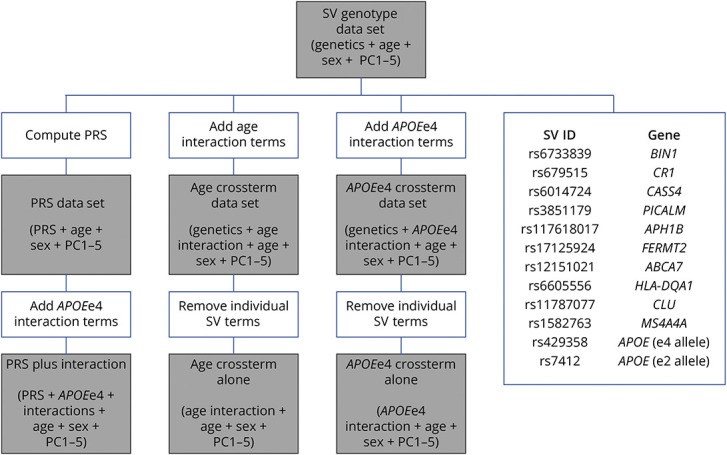
Flowchart of Data Set Creation for Model Testing Gray boxes are data sets, and white boxes are operations applied to create data sets.

To optimize the number of SVs under consideration for this proof-of-concept work, we focused on the top 20 independent SVs defined by having the largest effect sizes (i.e., largest Abs[Ln(odds ratio)]) showing genome-wide significant association with clinically diagnosed AD dementia in a recent large case/control GWAS.^5^ This choice allowed us to have few enough features (even after accounting for gene-gene interaction terms) that we could reasonably apply even the most data-intensive machine learning techniques. We also included *APOEe4* and *APOEe2* in this list given their well-known associations with amyloid levels in the population.^4,35,36^ Most machine learning methods struggle with sparse features (i.e., features that contain a large preponderance of zeros), so to mitigate this, SVs with a MAF <5% were removed. This resulted in a set of 12 SVs (listed in Figure 1) to be used for the rest of the study. Age, sex, and the first 5 genetic principal component eigenvectors were included as covariates in all models. A representation of all data sets created for this study is shown in Figure 1.

From this set of SVs, a standard PRS was computed according to the equation 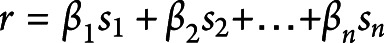, where *r* is risk score, β are effect sizes as determined by the GWAS (denoted by ln[odds ratio]), and s are dosage values for individual SVs. Linear regression was used to test the association of this AD PRS with amyloid PET burden, accounting for covariates specified earlier. In addition, interaction terms (as will be described shortly) were added as covariates for a separate regression. For ML model testing, a data set containing the genotype data for the 12 target SVs, covariates, and amyloid PET CL data was created.

As 1 aspect of the ML model approach, we used a novel quadratic encoding schema to consider the question of potential gene-gene interactions, shown in Equation 1
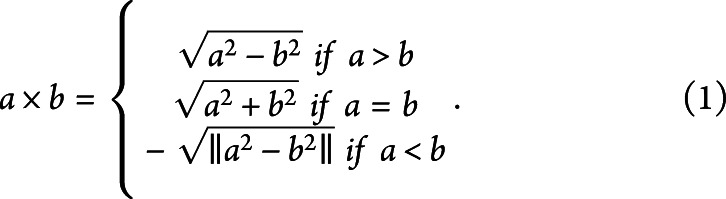
In this case, *a* and *b* are both dosage amounts for a given SV within the genotype data. This encoding method has an advantage over traditional coding methods because it preserves the relative dosage of the components in a way that standard methods would not^37^ and was used to create interaction terms for *APOEe4* with the other SVs used in this study. The interaction terms were then used to create a data set with the SV genotype data, interaction terms, covariates, and outcome. To observe whether information from these models was fully retained within the interaction terms themselves, we also created a comparator data set leaving out the individual SV genotypes themselves. A similar schema was used to assess SV-age interactions.

#### ML Model Selection and Training

We applied a series of ML models, each of which was trained on the same data set and for which the summary statistics of each model could be compared with select optimal performers. ML included in the stack were chosen primarily for known robustness to multicollinearity, which we expect to appear with the introduction of the derived gene-gene interaction terms. Several basic linear regressions were applied as part of the same pipeline for comparison with more complex ML models. These consisted of an ordinary least squares regression, a Lasso regression, an elastic net regression, and a partial least squares regression.

To test for potential nonlinear effects, tree-based models including a Random Forest, AdaBoost, and XGBoost were used to construct the other half of the model stack. Each of these works by taking many weak learners (such as basic decision trees or linear models) and combining them in algorithm-specific ways to generate a prediction. Because they are averaging across many weak learners, not all of which are linear, these models accounted for nonlinear effects in the genotype data. Finally, a stacked model was trained. This stacked model works by using the results of the 4 best previous models as features in a new regression, allowing interaction between model classes. All models except for XGBoost were sourced from the scikit-learn library.^38^ XGBoost was sourced from the xgboost package.^39^

To classify for amyloid PET status (negative vs positive) and cognitive status (unimpaired vs impaired), a separate stack of classification models was constructed. Because comparison between model types was not an outcome for the classification sets, model inclusion criteria were relaxed. Included models were logistic regression, decision tree, K-nearest neighbor, linear discriminant, quadratic discriminant, Gaussian naïve Bayes, support vector machines, stochastic gradient descent, random forest, gradient boosted trees, perceptron neural net, and a stacked model that functions similarly to the regression variant.

Twenty percent of the data was held out as a test set, and then models were trained and optimized on the remaining 80 percent using a grid search using five-fold cross-validation. Once trained, models were used to predict amyloid PET CL score on the test set, and the results of this final prediction are reported here. This process was repeated 100 times for each data set, with varying sets of points excluded in the test set, which allowed us to create a distribution for each of the metrics of interest instead of relying on a single report.

The r^2^ correlation between predicted and actual amyloid CL values were collected for each run. Tests were conducted for the normality of the r^2^ distribution to ensure that significance metrics are valid, and a two-sided independent *t* test was conducted to determine the significance of any separation between groups of models. For instances where predictions were binary categories, a receiver operating characteristic (ROC) curve was computed using class probabilities, and the area under the curve (AUC) was used as the outcome. Feature importances for models were computed using a permutation scheme where each feature was randomized, and then models were retrained on the data set with the randomized feature. Decrease in model r^2^ was the output of interest, with higher decrease meaning a more important feature.

### Standard Protocol Approvals, Registrations, and Patient Consents

All study protocols were approved by the Mayo Clinic and Olmsted Medical Center Institutional Review boards (for the MCSA) and by ADNI participating site Institutional Review Boards (for the ADNI). Written informed consent was obtained from all participants or their surrogates.

### Data Availability

Anonymized data will be available on reasonable request from a qualified investigator in accordance with the MCSA data-sharing protocol.

## Results

### Study Sample

Demographic information for the participants included in these analyses are summarized in the Table. The frequency of *APOEe4* positivity was lower in the population-based MCSA sample when compared with that of the ADNI sample, which was recruited in a manner similar to clinical trials. Mean education and amyloid PET burden were overall higher in ADNI participants compared with MCSA participants.

**Table:**
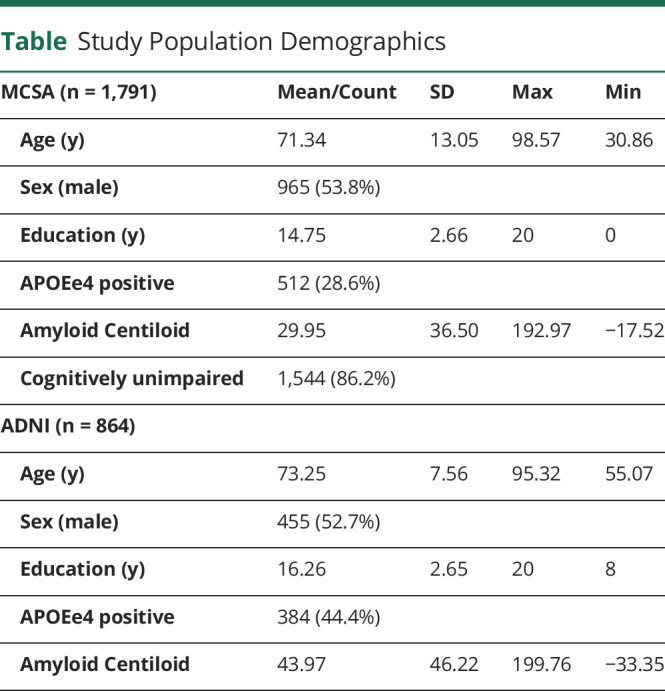
Study Population Demographics

MCSA (n = 1,791)	Mean/Count	SD	Max	Min
Age (y)	71.34	13.05	98.57	30.86
Sex (male)	965 (53.8%)			
Education (y)	14.75	2.66	20	0
APOEe4 positive	512 (28.6%)			
Amyloid Centiloid	29.95	36.50	192.97	−17.52
Cognitively unimpaired	1,544 (86.2%)			
ADNI (n = 864)				
Age (y)	73.25	7.56	95.32	55.07
Sex (male)	455 (52.7%)			
Education (y)	16.26	2.65	20	8
APOEe4 positive	384 (44.4%)			
Amyloid Centiloid	43.97	46.22	199.76	−33.35

### ML-PRS Outperforms Standard PRS in Predicting Amyloid PET Burden

**Figure 2:**
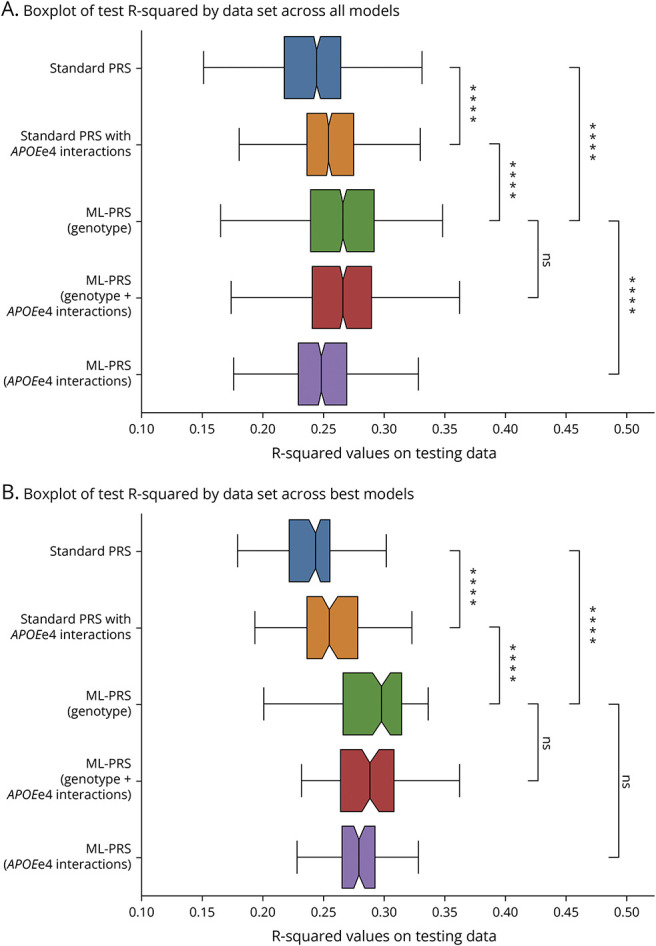
Performance of ML-PRS on Varying Data vs Standard PRS for Prediction of Amyloid PET Burden (A) shows the distribution across all model classes. All ML-PRS data sets perform significantly better than the PRS. (B) is the distribution for only the best model (determined by average *r*^2^ value across all data sets). Here, every other data set does significantly better than the PRS. Significance was determined with an independent two-sided *t* test. ns: 0.05 < *p*, *: 0.01 < *p* ≤ 0.05, **: 0.001 < *p* ≤ 0.01, ***: 0.0001 < *p* ≤ 0.001, ****: *p* ≤ 0.0001.

As shown in Figure 2, predicting amyloid CL value from trained ML-PRS models performed significantly better than a standard AD PRS generated from the same SVs (*p* < 0.0001). Specifically, compared with AD PRS [*r*^2^ = 0.242(0.241,0.244)], ML-PRS (specifically XGBoost based ML-PRS) explained a larger proportion of the variation in amyloid PET levels across the sample [*r*^2^ = 0.279(0.277,0.281)] on the test set. Both models (standard PRS and ML-PRS) included covariates. Even once gene-gene interaction terms were included as covariates in the standard PRS, ML-PRS continues to significantly outperform (*p* < 0.0001). This pattern held across individual ML models as well as the aggregate of all tested ML approaches.

### Nonlinear ML-PRS Models Perform Better than Linear Models

**Figure 3:**
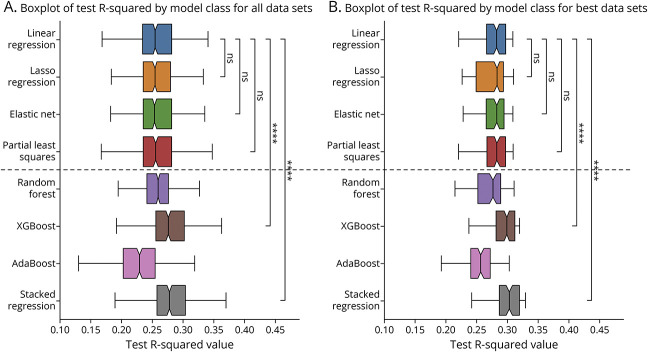
Performance of Linear vs Nonlinear ML-PRS Models for Genetic Risk Prediction All models above the dashed horizontal line are linear, whereas models below the line account for nonlinear effects. (A) shows distribution across all data sets. All linear models perform similarly, but there is variation in the nonlinear model types. (B) shows the distribution only for the singleton data set. In both cases, note that a meta-regressor (stacked regression) performs significantly better than any of the linear methods. Again, significance was determined through a two-sided independent *t* test. ns: 0.05 < *p*, *: 0.01 < *p* ≤ 0.05, **: 0.001 < *p* ≤ 0.01, ***: 0.0001 < *p* ≤ 0.001, ****: *p* ≤ 0.0001.

Linear ML models of all classes performed similarly to each other (Figure 3). Nonlinear ML-PRS models generally performed significantly better than linear ML-PRS models, particularly in considering a stacked regression of all approaches (*p* < 0.0001). There was variation in performance across nonlinear ML-PRS approaches. For example, AdaBoost, which relies on weak linear regressions as its foundation, underperformed compared with fully linear methods, while XGBoost, which uses small decision trees, showed significantly stronger performance.

### Addition of Gene-Gene Interaction Terms Provides Biological Insights

**Figure 4:**
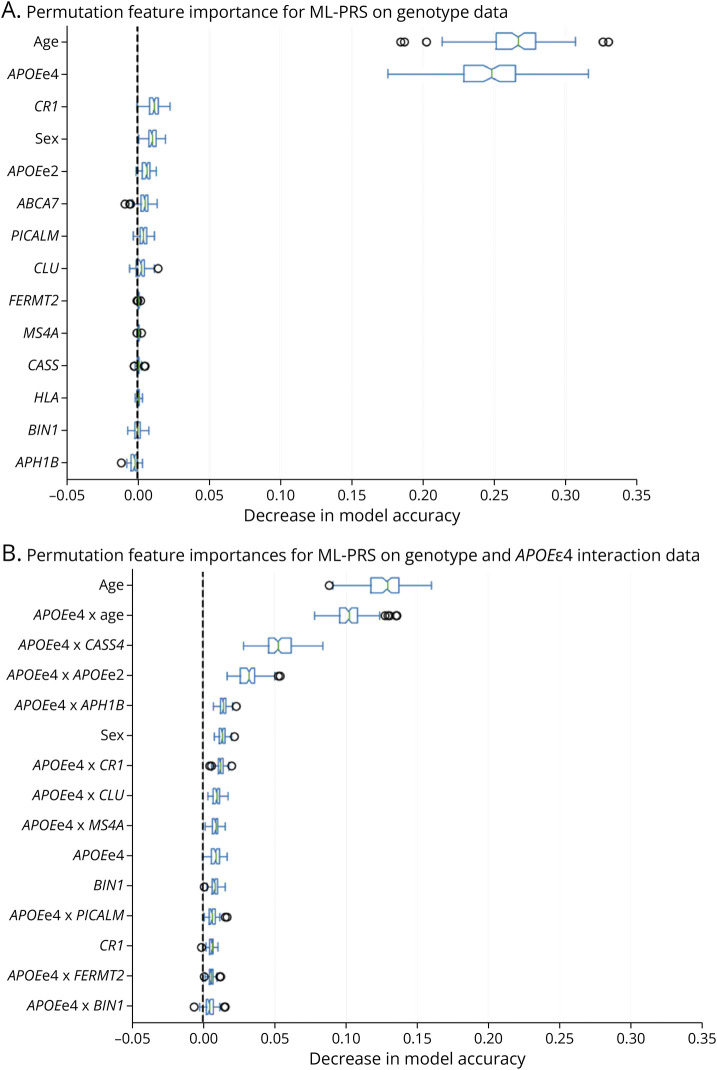
Relative Importances of Genetic and Demographic Factors Toward Predicting Amyloid PET Burden Higher values are more significant factors. (A) has feature importances for raw genotype data, while (B) provides importances including interactions with *APOEe4*. Note specifically the pairing of *APOEe4* and *CASS4*, which has notable importance when included as an interaction term, yet *CASS4* is not among the top 15 predictors on singleton data.

ML-PRS models based on both SV genotype data and SV-SV interaction terms overall performed better than the standard AD PRS but slightly worse than ML-PRS models without interaction terms (Figure 2). Removal of SV genotypes from these models (leaving the SV-SV interaction terms and covariates only) further degraded the *r*^2^ value, suggesting that not all the relevant information from individual genotypes is adequately captured within SV-SV interaction terms. Comparison of feature importance plots for the ML-PRS models without vs with interaction terms for *APOEe4* x other SVs yielded potentially relevant relationships (Figure 4). With genotype data alone, age and *APOEe4* were the dominant factors in prediction of amyloid PET burden, with sex, *CR1*, and *APOEe2* having more minor but nonzero influences (Figure 4A).

When *APOEe4* interaction terms were added for SV genotypes and age (Figure 4B), a different distribution of feature importances was observed. The influence of age was split between the age term alone and the *APOEe4* x age term, consistent with a meaningful interaction between these 2 factors on amyloidosis. Furthermore, the *APOEe4* genotype term was less important than 7 of the interaction terms between *APOEe4* and other risk alleles. Included among these was *CASS4* (cas scaffold protein family member 4), whose genotype alone was not a highly influential feature in the noninteraction model but where the interaction between *APOEe4* and *CASS4* was much more important. These statistical findings may suggest a potential biological relationship, akin to a “two-hit” model where the true effect of *APOEe4* on amyloid accumulation in the population overall may be distributed across subgroups where specific subcombinations of other AD risk variants are present.

### ML-PRS Methods Improve Classification of Amyloid PET Positivity and Cognitive Impairment vs Standard PRS

**Figure 5:**
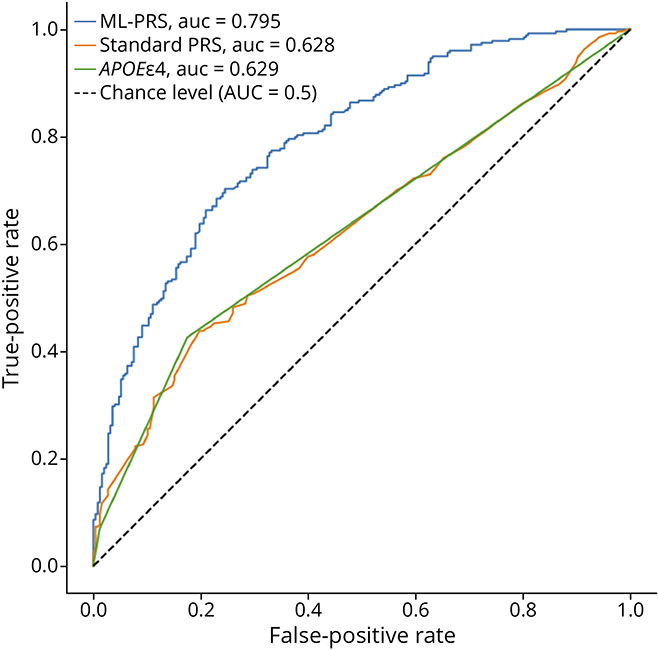
ROC Curves for Amyloid Case Status A Standard PRS performs on par with *APOEe4* alone and better than chance, but the ML-PRS model does better than either PRS or APOEe4. PRS = polygenic risk score; ROC = receiver operating characteristic.

**Figure 6:**
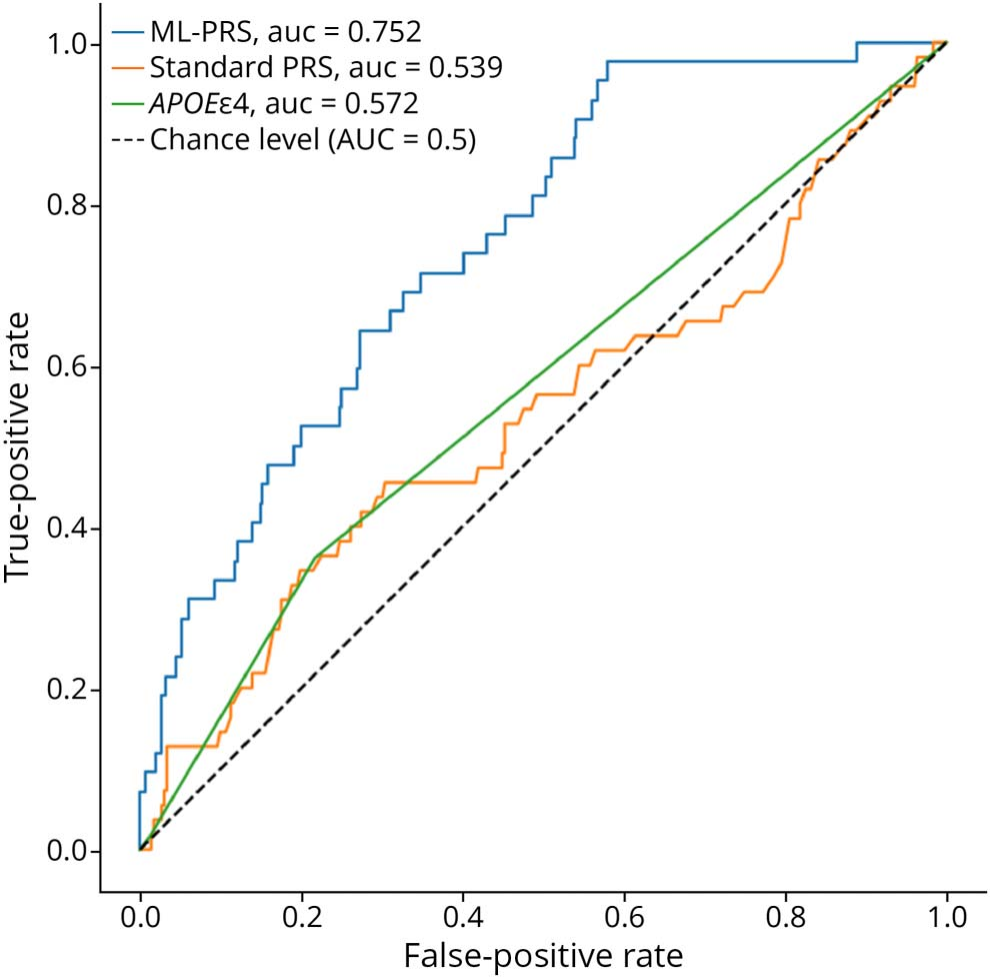
ROC Curves for Cognitively Unimpaired vs (MCI, AD) ML-PRS does better than either a standard PRS or *APOEe4* alone, both of which barely beat random chance for prediction. PRS = polygenic risk score; ROC = receiver operating characteristic.

We next assessed the predictive value of ML-PRS against other relevant AD outcomes. In classification of amyloid PET status (Figure 5), ML-PRS provides a better AUC (0.795) than *APOEe4* alone (AUC = 0.629) or the standard AD PRS including *APOEe4* and other risk variants (AUC = 0.628). Given that the variants analyzed in this study were drawn from GWAS of clinically diagnosed AD dementia, we also assessed the ML-PRS in predicting the presence of cognitive impairment (MCI or dementia vs cognitively unimpaired status). We observed that the ML-PRS (AUC = 0.752) outperformed *APOEe4* (AUC = 0.572) and a standard AD PRS (AUC = 0.539) in prediction of cognitive diagnosis (Figure 6). We observed that *APOEe4* and the standard PRS were both around chance levels in prediction of cognitive status impairment and that in fact the standard PRS was worse than *APOEe4* alone for this outcome. In both cases, the standard PRS displayed slightly degraded performance compared with *APOEe4* alone, but the differences are small and thus likely due to nuances of the specific small set of SVs chosen for this study.

## Discussion

We found that ML approaches performed better than standard PRS approaches for predicting amyloid PET burden (a key early biomarker of AD) based on risk variants for clinically diagnosed AD dementia. This improvement was present with both linear and nonlinear ML techniques but was particularly evident with ML methods that accounted for nonlinear genetic effects. These improvements persisted when using alternative AD-relevant phenotypes, including amyloid PET positivity and cognitive status (impaired vs unimpaired).

We had also hypothesized that inclusion of SV-SV interaction terms in our ML models would further improve their performance by explicitly accounting for epistatic effects within the genome. We did not find this to be the case because ML-PRS models with the addition of these interaction terms explained slightly less phenotypic variation than models based on genotype data alone (though the interaction-containing models still outperformed the standard PRS). However, ML-PRS methods using interaction terms allowed for further investigation of how the models (and their underlying genetic variants) realized their predictions. This is a particular advantage of ML methods over typical PRS approaches, the latter of which do not allow for the application of such post hoc explainer algorithms.

As an example, by comparing feature importance between models, we observed that the *CASS4-APOEe4* interaction term was highly important in model prediction compared with the relative importance of *CASS4* rs6014724 genotype in isolation. *CASS4* encodes a cell adhesion molecule important for axonal transport and has been implicated in *APP* (amyloid precursor protein) metabolism.^40,41^ Although *CASS4* was identified as a susceptibility gene for clinically diagnosed AD dementia, our results are illuminating toward a mechanistic explanation by specifically suggesting a relationship between *APOE* and *CASS4* on influencing amyloidosis. It is also highly plausible that genetic interaction effects are not limited to simple two-term SV × SV interactions. Instead, there may be more complex multi-SV combinations (e.g., 3–5 SVs) driving heterogeneous patterns of susceptibility to AD. These influences are not likely to be adequately captured by standard PRS approaches that amalgamate large combinations of variants across populations and therefore are likely to miss particular combination “hits,” which are driving disease at a precision level. Future work is planned extending our ML-PRS framework to these clustered combination scenarios.

Artificial intelligence approaches have been previously considered toward genetic risk prediction. A model of high-order Interactions-aware Polygenic Risk Score (hiPRS) has been proposed which uses a standard PRS as a base and then finds interactions of interest with Frequent Itemset Mining techniques.^42^ This approach was applied to risk of developing colorectal cancer, but in allowing interactions of any order to be selected, may miss interactions that are rare but highly impactful. Models from a published preprint attempted to predict clinically diagnosed late-onset AD through multifactor dimensionality reduction techniques, which break SV-SV combinations into high and low risk, and then test association with the phenotype.^43^ This approach, in reducing SV-SV combinations to a binary outcome, is limited by failing to capture the full variability of interactions possible between 2 SVs.

This work has limitations. The SVs chosen for use in this analysis were preselected from summary statistics for a published AD case/control GWAS, which is not a perfect proxy for biologically defined AD nor for amyloidosis as a key AD biomarker.^44^ In addition, to avoid complications to our ML approaches from data sparsity, we focused on a subset of common variants having strong effect sizes on clinically diagnosed AD dementia. Future extensions of this proof-of-concept work applying a broader list of input variants (including common and rare variants) may further improve performance.

We also elected to combine data from 2 cohorts (the MCSA and ADNI) to maximize statistical power for these analyses. However, these 2 data sets have different recruitment (population-based vs a structure similar to clinical trials), clinical composition (predominantly cognitively unimpaired MCSA participants vs majority MCI or dementia diagnoses in ADNI participants), and amyloid PET tracers used (PiB in the MCSA vs AV-45 in ADNI). Although we did not use a true external (i.e., independent from MCSA and ADNI) validation set, we did attempt to mitigate this limitation by using test data sets of MCSA/ADNI participants who were held out from the samples used to train the ML models. We also used the CL scale to harmonize (across MCSA and ADNI) the primary endophenotype used in these analyses, acknowledging that differences in the PET tracers and imaging protocols used in those 2 studies could still theoretically influence results when using a combined approach as in this work. In addition, our study was cross-sectional in design, reflecting the high clinical import of predicting susceptibility to amyloidosis (as a key early marker of AD pathophysiology and with emerging treatments targeting amyloid levels)^45^ and the sigmoidal nature of amyloid accumulation over time in the population (limiting the applicability of longitudinal change in amyloid burden as an endophenotype for this genetic work).^46^ However, extensions of the ML-PRS approach to other AD endophenotypes more amenable to a longitudinal study design (e.g., tau PET burden, cognition) represent natural future directions.

For AD and other complex diseases with genetic and lifestyle/environmental influences, the PRS approach has shown promise.^8,47^ There is clear attraction in the development of a blood-based summary measure that can be used in both the presymptomatic and symptomatic stages of disease (given that most elements of target genetic variation are present in both) for risk stratification. For genetic risk stratification of AD to have lasting clinical utility, predictive models will need to approach much higher accuracy (particularly for a disease presumed to have heritability between 60% and 80% based on twin studies^48^) than current AD PRS approaches achieve. Our analyses support that ML methods for genetic risk assessment may have greater value than standard PRS methods and therefore represent a high yield target in the development of a precision medicine approach to AD.

In summary, we show that ML methods applied to genetic data can do better than the standard PRS when predicting amyloid PET burden and other relevant AD phenotypes. Furthermore, methods that consider nonlinear genetic effects outperform linear methods, arguing for ML-PRS as an enhanced approach to capturing true genetic influences on a complex and heterogeneous disease. With further optimizations, our findings offer a path toward improved screening and risk stratification for AD.

## Appendix 1 Authors

**Table:**

Name	Location	Contribution
Nathaniel B. Gunter, BS/BA	Department of Radiology, Mayo Clinic Rochester	Drafting/revision of the article for content, including medical writing for content; study concept or design; and analysis or interpretation of data
Robel K. Gebre, PhD	Department of Radiology, Mayo Clinic Rochester	Drafting/revision of the article for content, including medical writing for content; study concept or design; and analysis or interpretation of data
Jonathan Graff-Radford, MD	Department of Neurology, Mayo Clinic Rochester	Drafting/revision of the article for content, including medical writing for content; major role in the acquisition of data
Michael G. Heckman, MS	Department of Quantitative Health Sciences, Mayo Clinic Florida	Drafting/revision of the article for content, including medical writing for content; study concept or design
Clifford R. Jack, Jr., MD	Department of Radiology, Mayo Clinic Rochester	Drafting/revision of the article for content, including medical writing for content; major role in the acquisition of data
Val J. Lowe, MD	Department of Radiology, Mayo Clinic Rochester	Drafting/revision of the article for content, including medical writing for content; major role in the acquisition of data
David S. Knopman, MD	Department of Neurology, Mayo Clinic Rochester	Drafting/revision of the article for content, including medical writing for content; major role in the acquisition of data
Ronald C. Petersen, MD, PhD	Department of Neurology; Department of Quantitative Health Sciences, Mayo Clinic Rochester	Drafting/revision of the article for content, including medical writing for content; major role in the acquisition of data
Owen A. Ross, PhD	Department of Neuroscience; Department of Clinical Genomics, Mayo Clinic Florida	Drafting/revision of the article for content, including medical writing for content; major role in the acquisition of data; and study concept or design
Prashanthi Vemuri, PhD	Department of Radiology, Mayo Clinic Rochester	Drafting/revision of the article for content, including medical writing for content; major role in the acquisition of data; study concept or design; and analysis or interpretation of data
Vijay K. Ramanan, MD, PhD	Department of Neurology, Mayo Clinic Rochester	Drafting/revision of the article for content, including medical writing for content; major role in the acquisition of data; study concept or design; and analysis or interpretation of data

## Appendix 2 Coinvestigators

**Table:**

Coinvestigators are listed at links.lww.com/NXG/A671.

## Glossary

AD: Alzheimer disease
ADNI: Alzheimer's Disease Neuroimaging Initiative
AUC: area under the curve
CL: Centiloid
GWAS: genome-wide association studies
MAF: minor allele frequency
MCSA: Mayo Clinic Study of Aging
ML: machine learning
PCA: principal component analysis
PiB: Pittsburgh compound B
PRS: polygenic risk score
SVs: sequence variants
SUVR: standardized uptake value ratio

## References

[1] Karlsson IK, Escott-Price V, Gatz M, et al. Measuring heritable contributions to Alzheimer's disease: polygenic risk score analysis with twins. Brain Commun. 2022;4(1):fcab308. doi:10.1093/braincomms/fcab308 35169705 PMC8833403

[2] Raghavan N, Tosto G. Genetics of Alzheimer's disease: the importance of polygenic and epistatic components. Curr Neurol Neurosci Rep. 2017;17(10):78. doi:10.1007/s11910-017-0787-1 28825204 PMC5699909

[3] Escott-Price V, Sims R, Bannister C, et al. Common polygenic variation enhances risk prediction for Alzheimer's disease. Brain. 2015;138(Pt 12):3673-3684. doi:10.1093/brain/awv268 26490334 PMC5006219

[4] Corder EH, Saunders AM, Strittmatter WJ, et al. Gene dose of apolipoprotein E type 4 allele and the risk of Alzheimer's disease in late onset families. Science. 1993;261(5123):921-923. doi:10.1126/science.8346443 8346443

[5] Bellenguez C, Küçükali F, Jansen IE, et al. New insights into the genetic etiology of Alzheimer's disease and related dementias. Nat Genet. 2022;54(4):412-436. doi:10.1038/s41588-022-01024-z 35379992 PMC9005347

[6] de Rojas I, Moreno-Grau S, Tesi N, et al. Common variants in Alzheimer's disease and risk stratification by polygenic risk scores. Nat Commun. 2021;12(1):3417. doi:10.1038/s41467-021-22491-8 34099642 PMC8184987

[7] Wightman DP, Jansen IE, Savage JE, et al. A genome-wide association study with 1,126,563 individuals identifies new risk loci for Alzheimer's disease. Nat Genet. 2021;53(9):1276-1282. doi:10.1038/s41588-021-00921-z 34493870 PMC10243600

[8] Chasioti D, Yan J, Nho K, Saykin AJ. Progress in polygenic composite scores in Alzheimer's and other complex diseases. Trends Genet. 2019;35(5):371-382. doi:10.1016/j.tig.2019.02.005 30922659 PMC6475476

[9] Leonenko G, Sims R, Shoai M, et al. Polygenic risk and hazard scores for Alzheimer's disease prediction. Ann Clin Transl Neurol. 2019;6(3):456-465. doi:10.1002/acn3.716 30911569 PMC6414493

[10] Desikan RS, Fan CC, Wang Y, et al. Genetic assessment of age-associated Alzheimer disease risk: development and validation of a polygenic hazard score. PLoS Med. 2017;14(3):e1002258. doi:10.1371/journal.pmed.1002258 28323831 PMC5360219

[11] Luckett ES, Abakkouy Y, Reinartz M, et al. Association of Alzheimer's disease polygenic risk scores with amyloid accumulation in cognitively intact older adults. Alzheimers Res Ther. 2022;14(1):138. doi:10.1186/s13195-022-01079-4 36151568 PMC9508733

[12] Jung S-H, Kim H-R, Chun MY, et al. Transferability of Alzheimer disease polygenic risk score across populations and its association with Alzheimer disease-related phenotypes. JAMA Netw Open. 2022;5(12):e2247162. doi:10.1001/jamanetworkopen.2022.47162 36520433 PMC9856322

[13] Kauppi K, Rönnlund M, Nordin Adolfsson A, Pudas S, Adolfsson R. Effects of polygenic risk for Alzheimer's disease on rate of cognitive decline in normal aging. Transl Psychiatry. 2020;10(1):250. doi:10.1038/s41398-020-00934-y 32709845 PMC7381667

[14] Ramanan VK, Heckman MG, Lesnick TG, et al. Tau polygenic risk scoring: a cost-effective aid for prognostic counseling in Alzheimer's disease. Acta Neuropathol. 2022;143(5):571-583. doi:10.1007/s00401-022-02419-2 35412102 PMC9109940

[15] Ramanan VK, Heckman MG, Przybelski SA, et al. Polygenic scores of Alzheimer's disease risk genes add only modestly to APOE in explaining variation in amyloid PET burden. J Alzheimers Dis. 2022;88(4):1615-1625. doi:10.3233/jad-220164 35811524 PMC9534315

[16] Wang H, Bennett DA, De Jager PL, Zhang Q-Y, Zhang H-Y. Genome-wide epistasis analysis for Alzheimer's disease and implications for genetic risk prediction. Alzheimers Res Ther. 2021;13(1):55. doi:10.1186/s13195-021-00794-8 33663605 PMC7934265

[17] Lo M-T, Kauppi K, Fan C-C, et al. Identification of genetic heterogeneity of Alzheimer's disease across age. Neurobiol Aging. 2019;84:243.e1-243.e9. doi. 10.1016/j.neurobiolaging.2019.02.022 PMC6783343 30979435

[18] Libbrecht MW, Noble WS. Machine learning applications in genetics and genomics. Nat Rev Genet. 2015;16(6):321-332. doi:10.1038/nrg3920 25948244 PMC5204302

[19] Reel PS, Reel S, Pearson E, Trucco E, Jefferson E. Using machine learning approaches for multi-omics data analysis: a review. Biotechnol Adv. 2021;49:107739. doi. 10.1016/j.biotechadv.2021.107739 33794304

[20] Elgart M, Lyons G, Romero-Brufau S, et al. Non-linear machine learning models incorporating SNPs and PRS improve polygenic prediction in diverse human populations. Commun Biol. 2022;5(1):856. doi:10.1038/s42003-022-03812-z 35995843 PMC9395509

[21] Roberts RO, Geda YE, Knopman DS, et al. The Mayo Clinic Study of Aging: design and sampling, participation, baseline measures and sample characteristics. Neuroepidemiology. 2008;30(1):58-69. doi:10.1159/000115751 18259084 PMC2821441

[22] Rocca WA, Yawn BP, St Sauver JL, Grossardt BR, Melton LJ, 3rd. History of the Rochester Epidemiology Project: half a century of medical records linkage in a US population. Mayo Clin Proc. 2012;87(12):1202-1213. doi:10.1016/j.mayocp.2012.08.012 23199802 PMC3541925

[23] Veitch DP, Weiner MW, Aisen PS, et al. Understanding disease progression and improving Alzheimer's disease clinical trials: recent highlights from the Alzheimer's Disease Neuroimaging Initiative. Alzheimers Dement. 2019;15(1):106-152. doi:10.1016/j.jalz.2018.08.005 30321505

[24] Weiner MW, Aisen PS, Jack CR, Jr., et al. The Alzheimer's disease neuroimaging initiative: progress report and future plans. Alzheimers Dement. 2010;6(3):202-211.e7. doi:10.1016/j.jalz.2010.03.007 20451868 PMC2927112

[25] Chang CC, Chow CC, Tellier LC, Vattikuti S, Purcell SM, Lee JJ. Second-generation PLINK: rising to the challenge of larger and richer datasets. Gigascience. 2015;4(1):7.doi:10.1186/s13742-015-0047-8 25722852 PMC4342193

[26] Das S, Forer L, Schonherr S, et al. Next-generation genotype imputation service and methods. Nat Genet. 2016;48(10):1284-1287. doi:10.1038/ng.3656 27571263 PMC5157836

[27] Taliun D, Harris DN, Kessler MD, et al. Sequencing of 53,831 diverse genomes from the NHLBI TOPMed Program. Nature. 2021;590(7845):290-299. doi:10.1038/s41586-021-03205-y 33568819 PMC7875770

[28] Ramanan VK, Lesnick TG, Przybelski SA, et al. Coping with brain amyloid: genetic heterogeneity and cognitive resilience to Alzheimer's pathophysiology. Acta Neuropathol Commun. 2021;9(1):48. doi:10.1186/s40478-021-01154-1 33757599 PMC7986461

[29] Saykin AJ, Shen L, Yao X, et al. Genetic studies of quantitative MCI and AD phenotypes in ADNI: progress, opportunities, and plans. Alzheimers Dement. 2015;11(7):792-814. doi:10.1016/j.jalz.2015.05.009 26194313 PMC4510473

[30] Jack CR Jr., Wiste HJ, Weigand SD, et al. Defining imaging biomarker cut points for brain aging and Alzheimer's disease. Alzheimers Demen. 2017;13(3):205-216. doi:10.1016/j.jalz.2016.08.005 PMC5344738 27697430

[31] Klunk WE, Engler H, Nordberg A, et al. Imaging brain amyloid in Alzheimer's disease with Pittsburgh Compound-B. Ann Neurol. 2004;55(3):306-319. doi. 10.1002/ana.20009 14991808

[32] Jagust WJ, Bandy D, Chen K, et al. The Alzheimer's disease neuroimaging initiative positron emission tomography core. Alzheimers Dement. 2010;6(3):221-229. doi:10.1016/j.jalz.2010.03.003 20451870 PMC2920531

[33] Klunk WE, Koeppe RA, Price JC, et al. The Centiloid Project: standardizing quantitative amyloid plaque estimation by PET. Alzheimers Dement. 2015;11(1):1-15.e1-4. doi:10.1016/j.jalz.2014.07.003 25443857 PMC4300247

[34] Collij LE, Salvadó G, Shekari M, et al. Visual assessment of [(18)F]flutemetamol PET images can detect early amyloid pathology and grade its extent. Eur J Nucl Med Mol Imaging. 2021;48(7):2169-2182. doi:10.1007/s00259-020-05174-2 33615397 PMC8175297

[35] Corder EH, Saunders AM, Risch NJ, et al. Protective effect of apolipoprotein E type 2 allele for late onset Alzheimer disease. Nat Genet. 1994;7(2):180-184. doi:10.1038/ng0694-180 7920638

[36] Ramanan VK, Risacher SL, Nho K, et al. APOE and BCHE as modulators of cerebral amyloid deposition: a florbetapir PET genome-wide association study. Mol Psychiatry. 2014;19(3):351-357. doi:10.1038/mp.2013.19 23419831 PMC3661739

[37] Gunter N, Vemuri P, Ramanan V, Gebre RK. A Novel Approach to Encode Two-Way Epistatic Interactions Between Single Nucleotide Polymorphisms. 2023:arXiv:2306.09175. Accessed June 1, 2023. ui.adsabs.harvard.edu/abs/2023arXiv230609175G.

[38] Pedregosa F, Varoquaux G, Gramfort A, et al. Scikit-learn: machine learning in Python. J Mach Learn Res. 2011;12:2825-2830.

[39] Chen T, Guestrin C. XGBoost: A Scalable Tree Boosting System. Presented at: Proceedings of the 22nd ACM SIGKDD International Conference on Knowledge Discovery and Data Mining; 2016; San Francisco, California, USA. doi:10.1145/2939672.2939785

[40] Dourlen P, Kilinc D, Malmanche N, Chapuis J, Lambert JC. The new genetic landscape of Alzheimer's disease: from amyloid cascade to genetically driven synaptic failure hypothesis? Acta Neuropathol. 2019;138(2):221-236. doi:10.1007/s00401-019-02004-0 30982098 PMC6660578

[41] Karch CM, Goate AM. Alzheimer's disease risk genes and mechanisms of disease pathogenesis. Biol Psychiatry. 2015;77(1):43-51. doi:10.1016/j.biopsych.2014.05.006 24951455 PMC4234692

[42] Massi MC, Franco NR, Manzoni A, et al. Learning high-order interactions for polygenic risk prediction. PLoS ONE. 2023;18(2):e0281618. doi:10.1371/journal.pone.0281618 36763605 PMC9916647

[43] Hermes S, Cady J, Armentrout S, et al. Epistatic features and machine learning improve Alzheimer's risk prediction over polygenic risk scores. medRxiv 2023:2023.02.10.23285766. doi:10.1101/2023.02.10.23285766 PMC11284654 38788065

[44] Therriault J, Pascoal TA, Benedet AL, et al. Frequency of biologically defined Alzheimer disease in relation to age, sex, APOE ε4, and cognitive impairment. Neurology. 2021;96(7):e975-e985. doi:10.1212/wnl.0000000000011416 33443136 PMC8055338

[45] Ramanan VK, Armstrong MJ, Choudhury P, et al. Antiamyloid monoclonal antibody therapy for Alzheimer disease: emerging issues in neurology. Neurology. 2023;101(19):842-852.doi:10.1212/wnl.0000000000207757 37495380 PMC10663011

[46] Jack CR, Jr., Wiste HJ, Lesnick TG, et al. Brain β-amyloid load approaches a plateau. Neurology. 2013;80(10):890-896. doi:10.1212/WNL.0b013e3182840bbe 23446680 PMC3653215

[47] Visscher PM, Yengo L, Cox NJ, Wray NR. Discovery and implications of polygenicity of common diseases. Science. 2021;373(6562):1468-1473. doi:10.1126/science.abi8206 34554790 PMC9945947

[48] Gatz M, Reynolds CA, Fratiglioni L, et al. Role of genes and environments for explaining Alzheimer disease. Arch Gen Psychiatry. 2006;63(2):168-174. doi:10.1001/archpsyc.63.2.168 16461860

